# Conventional Versus Traction-Assisted Endoscopic Submucosal Dissection for Esophageal Cancers: A Systematic Review and Meta-Analysis

**DOI:** 10.7759/cureus.113258

**Published:** 2026-07-23

**Authors:** Archit Garg, Muhammad T Bajwa, Aashi Garg, Aadhithyaraman Santharaman, Vishali Moond, Arkady Broder, Douglas G Adler

**Affiliations:** 1 Internal Medicine, Saint Peter's University Hospital/Robert Wood Johnson Medical School, New Brunswick, USA; 2 Medicine, Boston University School of Public Health, Boston, USA; 3 Gastroenterology, Saint Peter's University Hospital/Robert Wood Johnson Medical School, New Brunswick, USA; 4 Gastroenterology, West Virginia University, Morgantown, USA; 5 Gastroenterology and Hepatology, Saint Peter's University Hospital/Robert Wood Johnson Medical School, New Brunswick, USA; 6 Gastroenterology, Center for Advanced Therapeutic Endoscopy, Centura Health, Porter Adventist Hospital, Denver, USA

**Keywords:** advanced endoscopy, conventional endoscopic submucosal dissection, endoscopic submucosal dissection (esd), esophageal cancer (ec), traction-assisted endoscopic submucosal dissection

## Abstract

Endoscopic submucosal dissection (ESD) is widely used to treat esophageal cancer. Traction-assisted ESD (TA-ESD) has shown promising outcomes, potentially offering better clinical results than conventional ESD (C-ESD). This study compares the efficacy and safety of C-ESD versus TA-ESD in esophageal cancer treatment.

We conducted a meta-analysis of six studies involving 694 patients comparing clinical outcomes between the two techniques.

Mean dissection time was similar between TA-ESD and C-ESD (71.48 vs 72.02 minutes, p = 0.4). TA-ESD yielded a larger resected area (32.49 cm^2^ vs 25.05 cm^2^, p < 0.05). TA-ESD had higher curative resection rates, 52.5% (CI = 42.4-62.4; I^2^ = 97.8%), compared to C-ESD, 36.6% (CI = 25.4-49.5; I^2^ = 98.2%); RR 1.46 (CI = 1.15-1.84; p = 0.0018). En-bloc resection rates were 96.9% (CI = 90.9-98.7; I^2 ^= 19.3%) and 96.8% (CI = 82.5-99.5; I^2^ = 74.6%) with TA-ESD and C-ESD, respectively; RR 1.01 (CI = 0.96-1.09; p = 0.68). R0 resection rates with TA-ESD and C-ESD were 86.6% (CI = 62.3-96.2; I^2^ = 89.5%) and 77.6% (CI = 42.9-94.1; I^2^ = 95%), respectively; RR 1.11 (CI = 0.81-1.51; p = 0.28). Total adverse events with TA-ESD and C-ESD were 0.9% (CI = 0.2-3.6; I^2^ = 0%) and 4.1% (CI = 2.0-8.2; I^2^ = 95.9%), respectively; RR 0.08 (CI = 0.004-1.36; p = 0.08). Stricture development was more common with TA-ESD, 35.4% (CI = 24.3-48.4; I^2^ = 96.1%), compared to C-ESD, 19.5% (CI = 11.3-31.6; I^2^ = 97.5%); RR 1.33 (CI = 1.08-1.65; p = 0.0079). Perforation rates were lower with TA-ESD, 2.0% (CI = 0.5-7.2; I^2^ = 51.9%), compared to C-ESD, 3.6% (CI = 0.7-17.2; I^2^ = 85%), with RR 0.55 (CI = 0.32-0.94; p = 0.03).

TA-ESD demonstrated higher curative resection rates with larger resected area and lower perforation rates. However, it is associated with a higher risk of stricture formation. Further studies and cost-effective analysis are warranted to determine the optimal treatment approach.

## Introduction and background

Esophageal cancer can be squamous cell cancer, which predominates in the Asian population and typically arises in the mid-esophagus, while esophageal adenocarcinoma predominates in the Western population and arises at the gastroesophageal junction in the setting of Barrett's esophagus [1]. Endoscopic submucosal dissection (ESD) is widely used for treatment of superficial esophageal cancers and precancerous lesions [1]. The other endoscopic treatment option for these lesions is endoscopic mucosal resection (EMR). Recent studies have shown that ESD demonstrates superior en-bloc resection rates compared to EMR [1-3]. The resection size and shape can be more carefully controlled with ESD compared to EMR [2,4,5]. However, ESD is a more technically challenging procedure associated with longer procedural time and increased risk of adverse events compared to EMR [5,6].

Resection quality in ESD is best understood as a hierarchy of increasingly stringent endpoints. En-bloc resection refers to removal of a lesion as a single, non-fragmented piece, allowing accurate pathological staging, but it does not indicate complete tumor clearance. R0 resection refers to histological evidence of free vertical and horizontal margins from the resected en-bloc tumor specimen. Curative resection is the most stringent endpoint requiring R0 margins along with absence of poorly differentiated histology, lymph vascular invasion, and invasion beyond the superficial submucosa [1-4]. Moreover, curability by endoscopic resection is closely related to the depth of invasion, with lesions confined to the mucosa carrying low risk of lymph node invasion and can be managed with just curative endoscopic resection, whereas lesions invading the submucosa carry higher risk of lymph node spread requiring adjuvant therapy or esophagectomy irrespective of the resection technique used [4].

ESD for the treatment of early esophageal neoplasms can be particularly challenging due to the narrow lumen and thinner wall of the esophagus [7,8]. As the esophageal ESD proceeds, the mobility of the lesion also increases, with the lesion moving towards the anorectal region, which makes visualization difficult [9]. Hence, esophageal ESD requires more technical expertise and is associated with prolonged procedural times compared to ESD at other locations [10-12]. Moreover, esophageal ESD has been associated with adverse events including perforation, bleeding, pneumomediastinum, and mediastinitis [13,14]. To overcome these problems, traction techniques have been developed to increase exposure, reduce tissue mobility, and potentially make ESD both faster and technically easier.

Traction-associated ESD (TA-ESD) has been increasingly used [10,15]. TA-ESD involves the application of countertraction after dissecting the mucosa, which increases separation between tissue planes and facilitates better visualization of the space between the mucosa and deeper muscle layer [10]. Traction promotes more efficient tissue dissection, which, in turn, can contribute toward shortening procedure duration. Traction can be applied by various techniques including clips with thread, traction clips, ring-shaped thread, and forceps. Studies have shown increased efficacy with fewer adverse events associated with TA-ESD, although results have been limited by small sample sizes [16,17].

With the widespread adoption of both techniques and the growing volume of published data, there is a need for a comprehensive comparison between the two. There is no reported meta-analysis comparing TA-ESD and C-ESD specifically for esophageal cancers. We conducted a meta-analysis to determine the pooled rates of efficacy and safety of TA-ESD vs C-ESD in the treatment of esophageal cancers.

This article was previously presented as a meeting abstract at the 2025 Digestive Disease Week (DDW) Annual Scientific Meeting on May 3, 2025 [18].

## Review

Methods

We followed the PRISMA (Preferred Reporting Items for Systematic Reviews and Meta-Analyses) guidelines and the MOOSE (Meta-Analysis of Observational Studies in Epidemiology) checklist [19]. This systematic review was not registered in any registry (e.g., PROSPERO).

Search Strategy

We systematically searched PubMed, EMBASE, Google Scholar, LILACS (Latin American and Caribbean Health Sciences Literature), SCOPUS, and Web of Science from each database’s inception to April 05, 2025.

The literature search utilized combinations of the following keywords: “endoscopic submucosal dissection,” “conventional endoscopic submucosal dissection,” “traction assisted endoscopic submucosal dissection,” “traction endoscopic submucosal dissection”, “ESD,” “C-ESD,” “T-ESD,” “TA-ESD,” and “esophageal cancer.” Studies were limited to those involving human subjects, published in English, and appearing in peer-reviewed journals. Two authors (AG and MB) independently screened the titles and abstracts identified in the primary search, excluding studies that did not meet the research question based on predefined inclusion and exclusion criteria. The full texts of remaining articles were assessed for relevance. Any disagreements regarding article selection were resolved by consensus or discussion with a third author (DGA).

As this review was not prospectively registered and the search was limited to English-language publications, some relevant non-English studies may not have been captured. This is discussed in the Limitations section.

We also manually reviewed the reference lists of selected articles, as well as relevant systematic and narrative reviews, to identify additional pertinent studies.

Study Selection

We included both retrospective cohort and prospective randomized studies that reported clinical outcomes of TA-ESD compared to C-ESD for esophageal cancer, provided they met the following criteria: (1) participants diagnosed with esophageal cancer, regardless of age; (2) either TA-ESD or C-ESD was used as an intervention; (3) outcomes for TA-ESD and C-ESD were compared within the same study; and (4) results were reported for en-bloc resection, R0 resection, curative resection, mean operation time, bleeding rates, recurrence rates, and complication rates (including pneumonia, mediastinitis, perforation, stricture development, and muscle injury). Studies were included regardless of geographical location or whether they were abstracts or full manuscripts, as long as adequate data for analysis were provided. We excluded studies that (1) did not provide sufficient data to assess the outcomes of interest or (2) used interventions other than TA-ESD or C-ESD. For multiple publications from the same cohort, only the most recent and comprehensive report was included.

Data Abstraction and Quality Assessment

Three authors (AG, MTB, and AS) independently extracted data on study-related outcomes from each included study using a standardized form. Two authors (AG and MTB) independently assessed study quality using the Newcastle-Ottawa scale for retrospective cohort studies [20].

Outcomes Assessed

The primary outcome of this meta-analysis was pooled curative resection, as this represented the most clinically meaningful measure of oncologic adequacy in esophageal ESD. Secondary outcomes included mean resection area, mean dissection time, pooled en-bloc resection rates, pooled R0 resection rates, pooled rates of adverse events, pooled perforation rates, pooled postoperative bleeding rates, pooled rates of incidence of pneumonia, pooled rates of incidence of mediastinitis, pooled rates of muscle injury, pooled stricture development rates, and pooled recurrence rates associated with TA-ESD and C-ESD. Each outcome-specific pooled analysis was performed using only those studies that reported the respective endpoint. Studies lacking data for a given outcome were excluded from that particular analysis but were retained for outcomes they did report.

The mean dissection time/procedure duration was defined as the time taken from marking the lesion to complete resection of the tumor. Mean resected area was calculated by multiplying the maximum horizontal and vertical diameters of the resected mass. En-bloc resection was defined as resection of the tumor as a single piece. R0 resection was defined as absence of lateral or vertical margin involvement in en-bloc resected tumors. Curative resection was defined as R0 resection, absence of poorly differentiated or undifferentiated tumor, no lymph node or vascular invasion, and restriction to the superficial submucosa. Perforation was defined as the mediastinum being visualized during the operative intervention or postoperative chest imaging findings of mediastinal air/emphysema. Postoperative bleeding rates were defined by clinical (hematemesis/melena) or laboratory evidence of bleeding during the postoperative period. Mediastinitis and pneumonia were defined based on clinical and radiographic evidence. Stricture development was defined as luminal narrowing that cannot be traversed through a standard endoscope. Recurrence was defined as the return of tumor in the esophagus, lymph nodes, or distant organs following intervention. Adverse events and their severity were classified based on the American Society of Gastrointestinal Endoscopy (ASGE) Lexicon [21].

Statistical Analysis

We performed meta-analyses to calculate pooled estimates for each outcome, following the random-effects model described by DerSimonian and Laird [22], with effect sizes measured as risk probabilities. For studies reporting zero incidence of an outcome, we applied a continuity correction by adding 0.01 to the number of incident cases before statistical analysis [23]. Heterogeneity among study-specific estimates was evaluated using the Cochrane Q and I² statistics [24]. We also calculated the 95% prediction interval to address the dispersion of effect sizes [25,26]. Publication bias was assessed qualitatively by visual inspection of funnel plots and quantitatively using Egger’s test. All analyses were conducted using Comprehensive Meta-Analysis (CMA) software, version 4 (BioStat, Englewood, NJ).

Continuous outcomes presented as medians with minimum and maximum values, 95% confidence intervals, or interquartile ranges (IQR) were converted to means using the approach proposed by Luo et al. [27]. The corresponding standard deviations (SD) were determined using the method described by Wan et al. [28].

Results

Search Results and Population Characteristics

**Figure 1:**
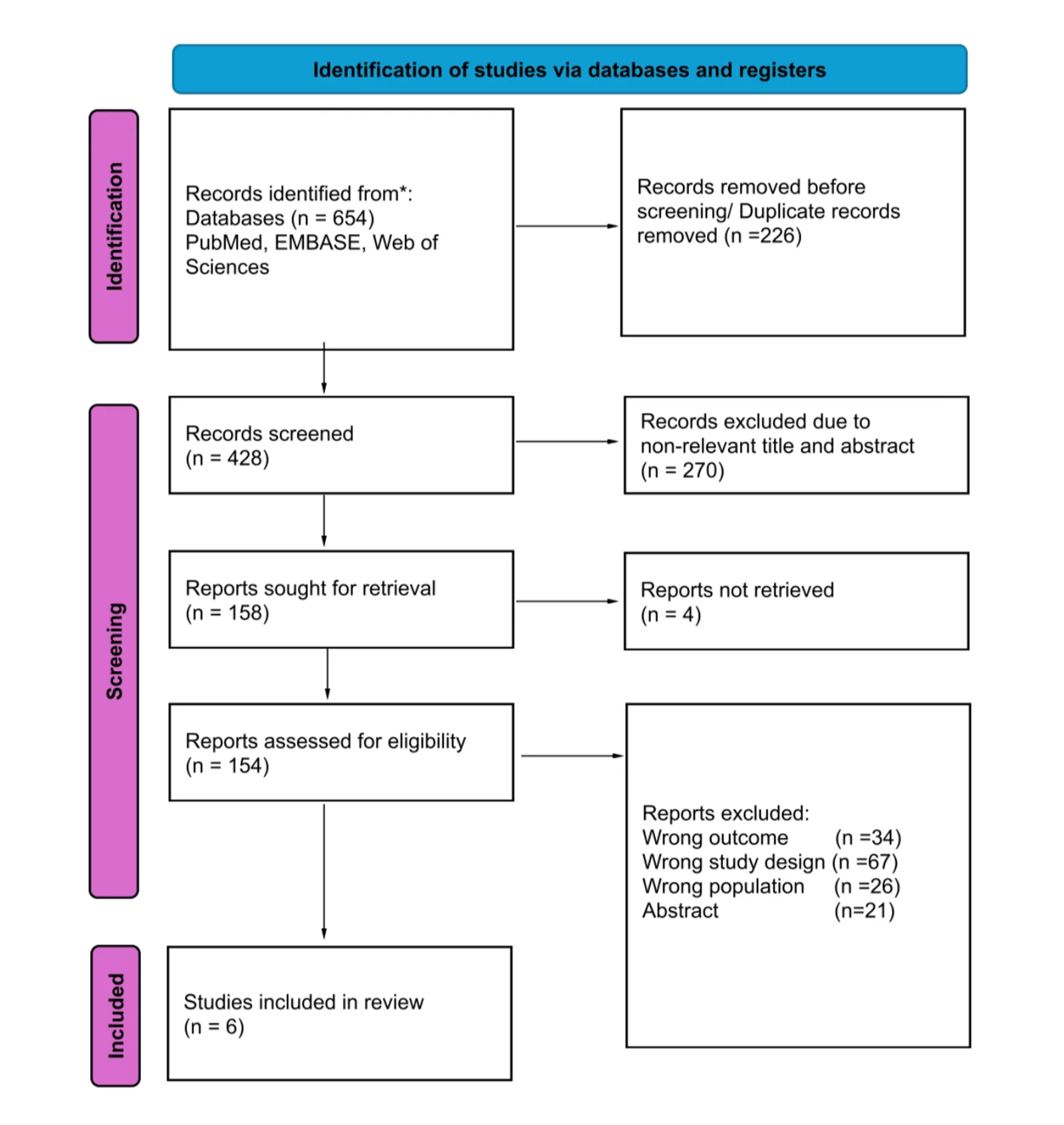
PRISMA study selection flow chart

Out of 654 initial records, 428 titles were screened, and 154 articles underwent full-text review. Of these, 148 studies were excluded for not meeting the inclusion criteria, leaving 6 full-text studies for the final analysis [16,17,29-32]. A schematic diagram of the study selection process is shown in Figure 1.

Among the six included studies, two were prospective, and four were retrospective, encompassing a total of 694 subjects. Of these, 351 patients (81.48% male; mean age 67.97 years) underwent TA-ESD, while 343 patients (74.64% male; mean age 68.32 years) received C-ESD. The population characteristics are presented in Table 1.

**Table 1:** Baseline population characteristics TA-ESD: Traction-assisted endoscopic submucosal dissection, C-ESD: Conventional endoscopic submucosal dissection, RCT: Randomized controlled trial, UT: Upper thoracic esophagus, MT: Middle thoracic esophagus, LT: Lower thoracic esophagus, GE: Gastroesophageal junction, AE: Abdominal esophagus, EP: Carcinoma in situ, LPM: Lamina propria mucosa, MM: Muscularis mucosa, SM1: Upper third of the submucosal layer.

Study	Study Details	Total Patients		Age (years)		Sex (Male/Female)		Tumor Max Diameter (mm)		Tumor Site		Tumor Circumferential Involvement (<1/2, ≥1/2)		Macroscopic Type (IIa, IIb, IIc)		Histological Depth of Invasion (EP, LPM, MM, SM1, and SM2)	
		TA-ESD	C-ESD	TA-ESD	C-ESD	TA-ESD	C-ESD	TA-ESD	C-ESD	TA-ESD	C-ESD	TA-ESD	C-ESD	TA-ESD	C-ESD	TA-ESD	C-ESD
Ota et al., 2012 [31]	Retrospective cohort study, single center, 2005-2010, Japan	67	20	68.2	66.2	61/6	17/3	28.1	26.4	-	-	<1/2 52/67 (77.6%), ≥1/2 15/67 (22.4%)	<1/2 16/20 (80%), ≥1/2 5/20 (20%)	-	-	EP 21/67 (31.3%), LPM 27/67 (40.3%), MM 11/67 (16.4%), SM1 8/67 (11.9%)	EP 10/20 (50%), LPM 3/20 (15%), MM 2/20 (10%), SM1 5/20 (25%)
Koike et al., 2015 [17]	RCT, single center, May 2012-Feb 2013, Japan	20	20	71.0 ± 6.3	69.5 ± 9.5	18/2	12/8	24.0 (11–92)	27.0 (8–48)	UT 4/20 (20%), MT 14/20 (70%), LT 2/20 (10%)	UT 1/20 (5%), MT 13/20 (65%), LT 6/20 (30%)	<1/2 12/20 (60%), ≥1/2 8/20 (40%)	<1/2 13/20 (65%), ≥ 1/2 7/20 (35%)	IIa 2/20, IIb 5/20, IIc 13/20	IIa 1/20, IIb 5/20, IIc 14/20	EP 9/20 (45%), LPM 5/20 (25%), MM 2/20 (10%), SM1 0/20 (0%), SM2 4/20 (20%)	EP 10/20 (50%), LPM 3/20 (15%), MM 2/20 (10%), SM1 5/20 (25%)
Xie et al., 2017 [32]	Retrospective study, single center, March 2014-June 2015, China	50	50	61.00 ± 8.12	63.46 ± 8.91	35/15	37/13	40	43	UT 4/50 (8.0%), MT 39/50 (78.0%), LT 7/50 (14.0%)	UT 1/50 (2.0%), MT 43/50 (86.0%), LT 6/50 (12.0%)	<1/2 20/50 (40.0%), ≥1/2 30/50 (60.0%)	<1/2 26/50 (52.0%), ≥1/2 24/50 (48.0%)	IIa 3/50 (6%), IIb 46/50 (92%), IIc 1/50 (2%)	IIa 9/50 (18%), IIb 41/50 (82%), IIc 0/50 (0%)	-	-
Yoshida et al., 2020 [16]	RCT, multicenter, October 2016-March 2019, Japan	116	117	70 (41–86)	72 (51–88)	101/15	95/22	30 (20–80)	30 (20–110)	UT 12/116 (10.3%), MT 74/116 (63.8%), LT 29/116 (25.0%), AE 1/116 (0.9%)	UT 13/117 (11.1%), MT 70/117 (59.8%), LT 30/117 (25.6%), AE 4/117 (3.4%)	<1/2 75/116 (64.7%), ≥1/2-1 35/116 (30.2%), full involvement 6/116 (5.2%)	<1/2 75/117 (64.1%), ≥1/2-1 37/117 (31.6%), full involvement 5/117 (4.3%)	Elevated (0-I, 0–IIa) 4/116 (3.4%), depressed (0–IIb, 0–IIc, 0–III) 108/116 (93.1%), mixed (0-lla+llc, 0-llc+lla, 0-I+IIa) 4/116 (3.4%)	Elevated (0-I, 0–IIa) 5/117 (4.3%), depressed (0–IIb, 0–IIc, 0–III) 107/117 (91.5%), mixed (0-lla+llc, 0-llc+lla, 0-I+IIa) 5/117 (4.3%)	EP 20/116 (17.2%), LPM 72/116 (62.1%), MM 20/116 (17.2%), SM1 4/116 (3.4%)	EP 16/117 (13.7%), LPM 81/117 (69.2%), MM 16/117 (13.7%), SM1 4/117 (3.4%)
Dai et al., 2024 [29]	Retrospective cohort study, single center, December 2017-February 2023, China	35	33	66.63 ± 6.61	66.75 ± 6.55	18/17	16/17	42.8 ± 13.5	44.2 ± 15.3	UT 3/35 (8.6%), MT 25/35 (71.4%), LT 7/35 (20.0%)	UT 0/33 (0%), MT 25/33 (75.8%), LT 8/33 (24.2%)	-	-	-	-	M1 17/35 (48.6%), M2 6/35 (17.1%), M3 8/35 (22.9%), SM1 4/35 (11.4%)	M1 14/33 (42.4%), M2 6/33 (18.2%), M3 5/33 (15.6%), SM1 8/33 (24.2%)
Joseph et al., 2024 [30]	Retrospective cohort study, multicenter, January 01, 2013-January 01, 2023, Brazil and USA	63	103	71.0 (65-78)	72.0 (63.0-78)	53/10	79/24	29.0 (20.0-35.5)	28.0 (20.0-37.5)	UT 0/63 (0%), MT 4/63 (6.3%), LT 37/63 (58.7%), GE Junction 22/63 (34.9%)	UT 2/103 (2.1%), MT 24/103 (25.3%), LT 36/103 (37.9%), GE Junction 33/103 (34.7%)	-	-	-	-	-	

Characteristics and Quality of Included Studies

None of these studies were population-based. All were original research articles. Each study provided detailed information on eradication rates and clinical outcomes, and importantly, there were no reports of patient loss to follow-up. Regarding methodological rigor, two studies were rated as high quality and four as medium quality, with none classified as low quality. Study quality assessments are summarized in Table 2.

**Table 2:** Study quality assessment (Newcastle-Ottawa scale)

Study	Selection				Comparability	Outcome			Score	Quality
	Representativeness of the average adult in the community	Cohort size	Information on clinical outcomes	Outcome not present at start	Factors comparable between the groups	Adequate clinical assessment	Follow-up time	Adequacy of follow-up	Max = 8	High > 6, medium: 4 to 6, low < 4
	Population based: 1; Multicenter: 0.5; Single-center: 0	>40 patients: 1; 39 to 20: 0.5; <20: 0	Information with clarity: 1; Information derived from percentage value: 0.5; unclear: 0	Not present: 1; present: 0	Yes: 1; no: 0	Yes: 1; no: 0	Yes: 1; not mentioned: 0	*All patients followed up: 1; >50% followed up: 0.5; <50% followed up or not* mentioned: 0		
Yoshida et al., 2020 [16]	0.5	1	1	1	NA	1	1	1	6.5	High
Koike et al., 2015 [17]	0	1	1	1	NA	1	1	1	6	Medium
Dai et al., 2024 [29]	0	1	1	1	NA	1	1	1	6	Medium
Joseph et al., 2024 [30]	0.5	1	1	1	NA	1	1	1	6.5	High
Ota et al., 2012 [31]	0	1	1	1	NA	1	1	1	6	Medium
Xie et al., 2017 [32]	0	1	1	1	NA	1	1	1	6	Medium

Three authors (AG, MTB, and AS) independently extracted data on study-related outcomes from each included study using a standardized form. Two authors (AG and MTB) independently assessed study quality using the Newcastle-Ottawa scale for retrospective cohort studies [20].

Meta-Analysis Outcomes

**Figure 2:**
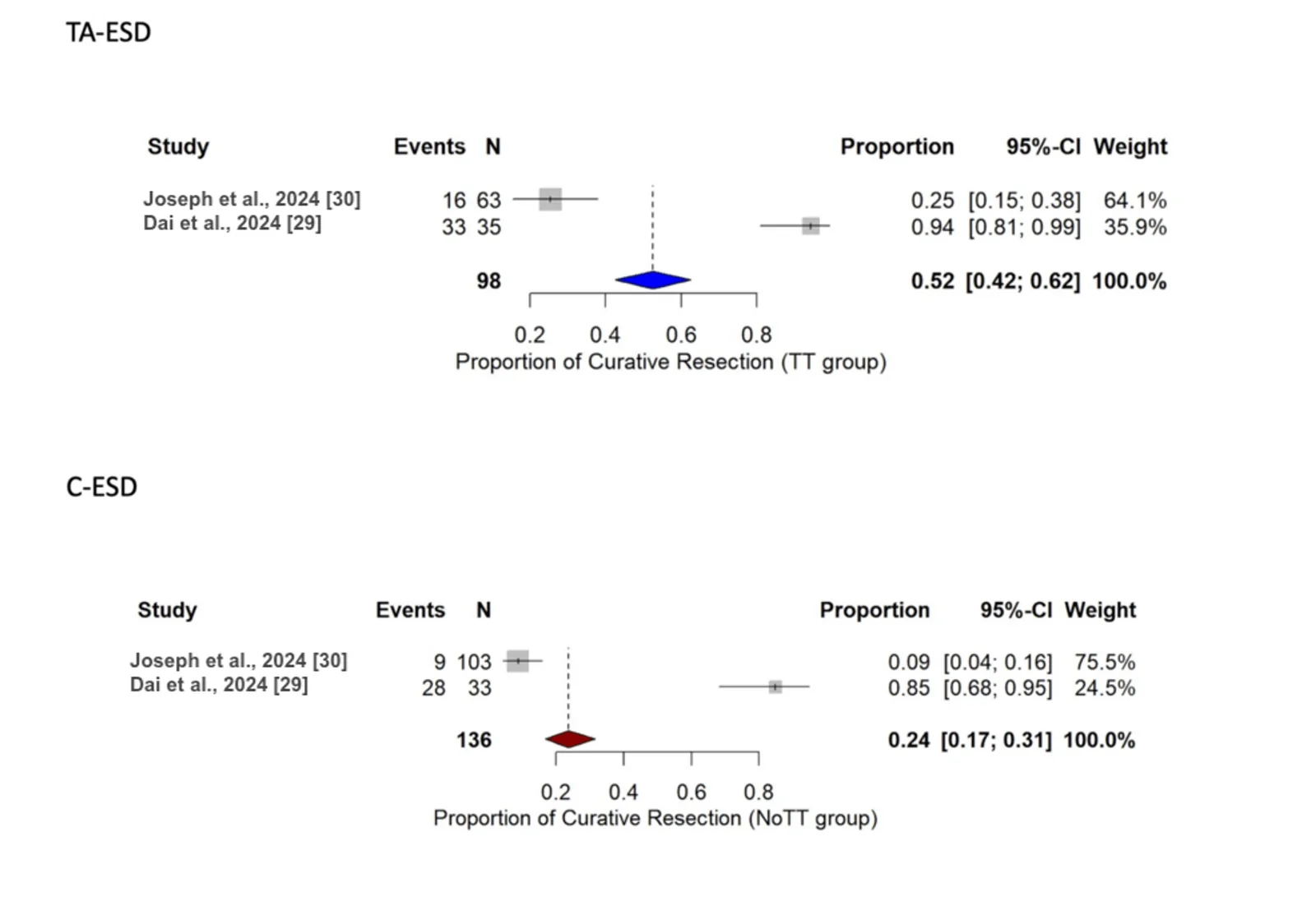
Forest plot, pooled rates of curative resection of TA-ESD vs C-ESD in esophageal cancer TA-ESD: Traction-assisted endoscopic submucosal dissection; C-ESD: Conventional endoscopic submucosal dissection.

**Figure 3:**
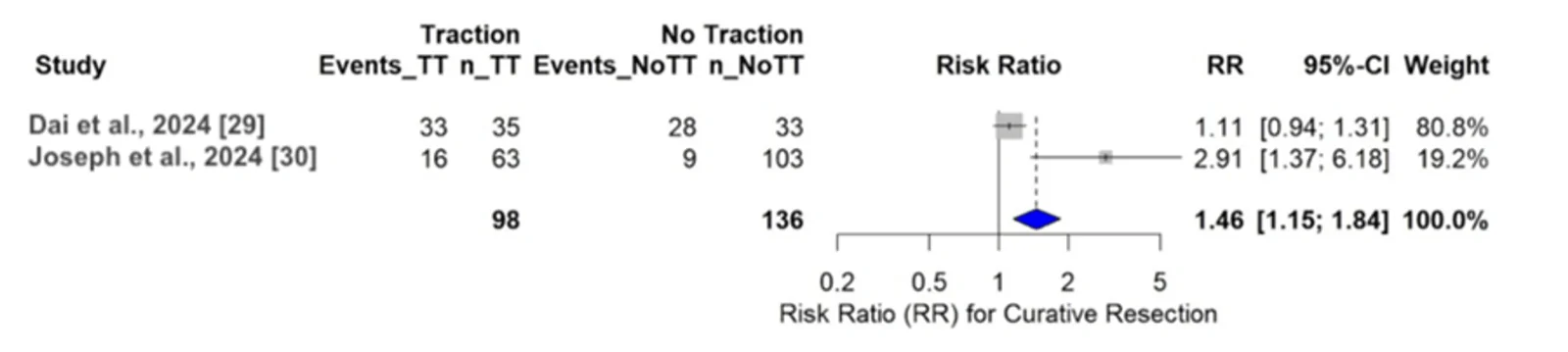
Forest plot, RR of curative resection of TA-ESD vs C-ESD in esophageal cancer TA-ESD: Traction-assisted endoscopic submucosal dissection; C-ESD: Conventional endoscopic submucosal dissection.

Curative resection: Two studies reported curative resection. The pooled curative resection rates were significantly higher with TA-ESD, 52.5% (CI = 42.4-62.4; I^2^ = 97.8%), in comparison to C-ESD, 36.6% (CI = 25.4-49.5; I^2^ = 98.2%), with RR 1.46 (CI = 1.15-1.84; p = 0.0018). These results should therefore be interpreted with considerable caution. The corresponding forest plot for pooled rates and RR is shown in Figures 2, 3, respectively.

En-bloc resection: In the analysis, three studies reported the en-bloc resection rates. The pooled en-bloc resection rate was 96.9% (CI = 90.9-98.7; I^2^ = 19.3%) with TA-ESD and 96.8% (CI = 82.5-99.5; I^2^ = 74.6%) with C-ESD, with RR 1.01 (CI = 0.96-1.09; p = 0.68).

R0 resection: Of the 234 patients for whom R0 resection was reported (three studies), the pooled R0 resection rate associated with TA-ESD and C-ESD was 86.6% (CI = 62.3-96.2; I^2^ = 89.5%) and 77.6% (CI = 42.9-94.1; I^2^ = 95%), respectively; RR 1.11 (CI = 0.81-1.51; p = 0.28).

Mean dissection time: Mean dissection time was 71.48 ± 49.63 minutes with TA-ESD and 72.02 ± 31.52 minutes with C-ESD, with a p-value of 0.4.

Mean resection area: The mean resected area of the tumor was greater in patients who underwent TA-ESD, 32.49 (21.98-43.0) cm^2^, in comparison to patients who underwent C-ESD, 25.05 (24.0-26.1) cm^2^, with a p-value of < 0.05.

**Figure 4:**
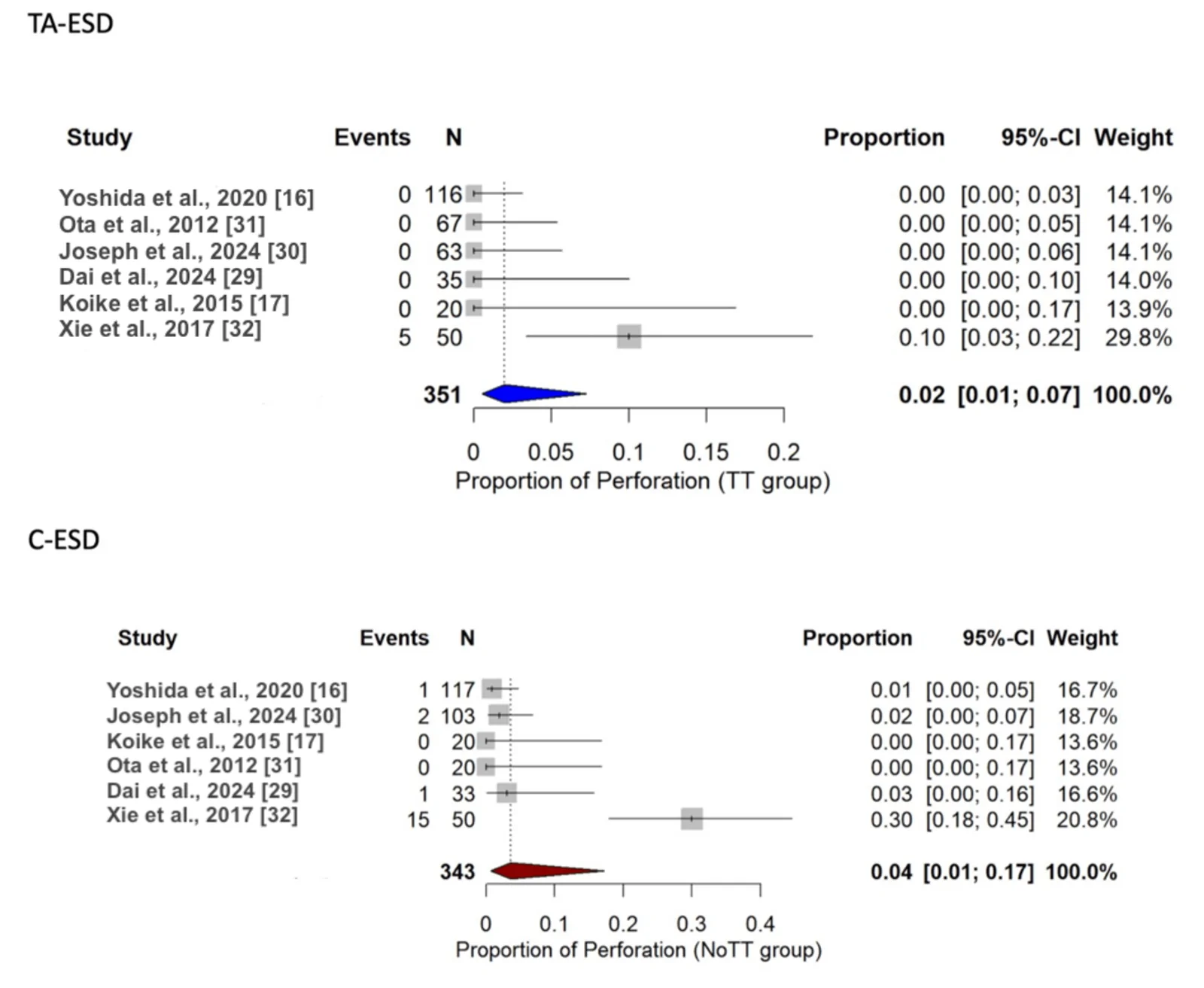
Forest plot, pooled rates of adverse events of TA-ESD vs C-ESD in esophageal cancer TA-ESD: Traction-assisted endoscopic submucosal dissection; C-ESD: Conventional endoscopic submucosal dissection.

**Figure 5:**
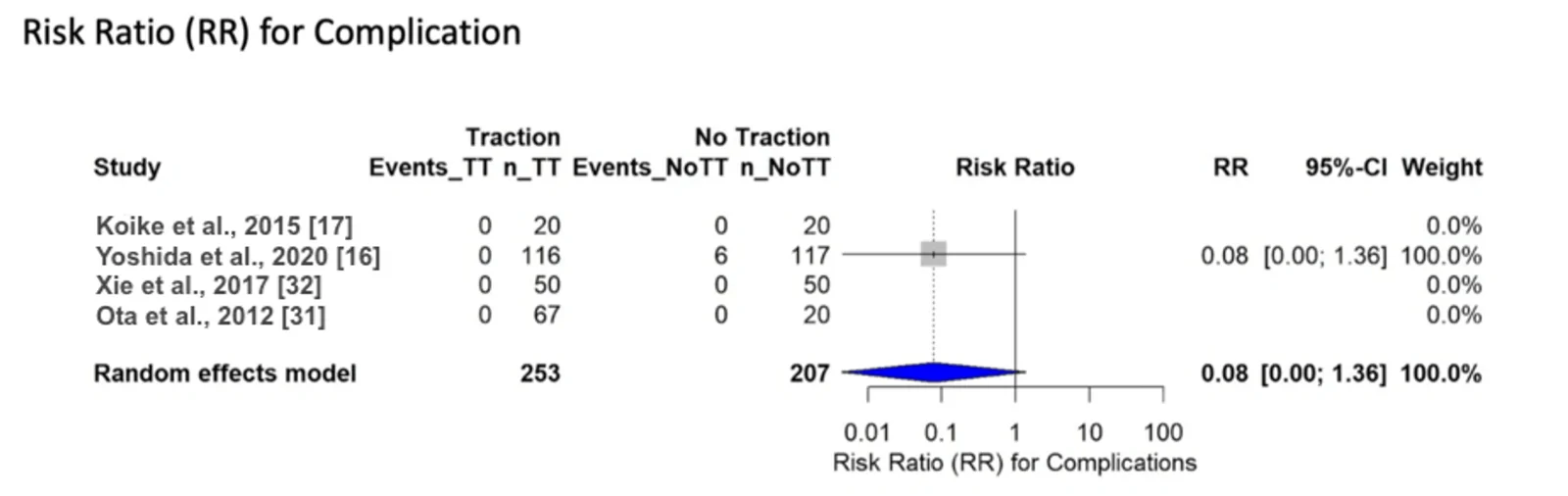
Forest plot, RR of adverse events of TA-ESD vs C-ESD in esophageal cancer TA-ESD: Traction-assisted endoscopic submucosal dissection; C-ESD: Conventional endoscopic submucosal dissection.

Total adverse events: The total adverse event rates associated with TA-ESD and C-ESD were 0.9% (CI = 0.2-3.6; I^2^ = 0%) and 4.1% (CI = 2.0-8.2; I^2^ = 0%), respectively, with RR 0.08 (CI = 0.004-1.36; p = 0.08). The corresponding forest plot for pooled rates is shown in Figure 4, and the corresponding forest plots for RR are shown in Figure 5, respectively.

**Figure 6:**
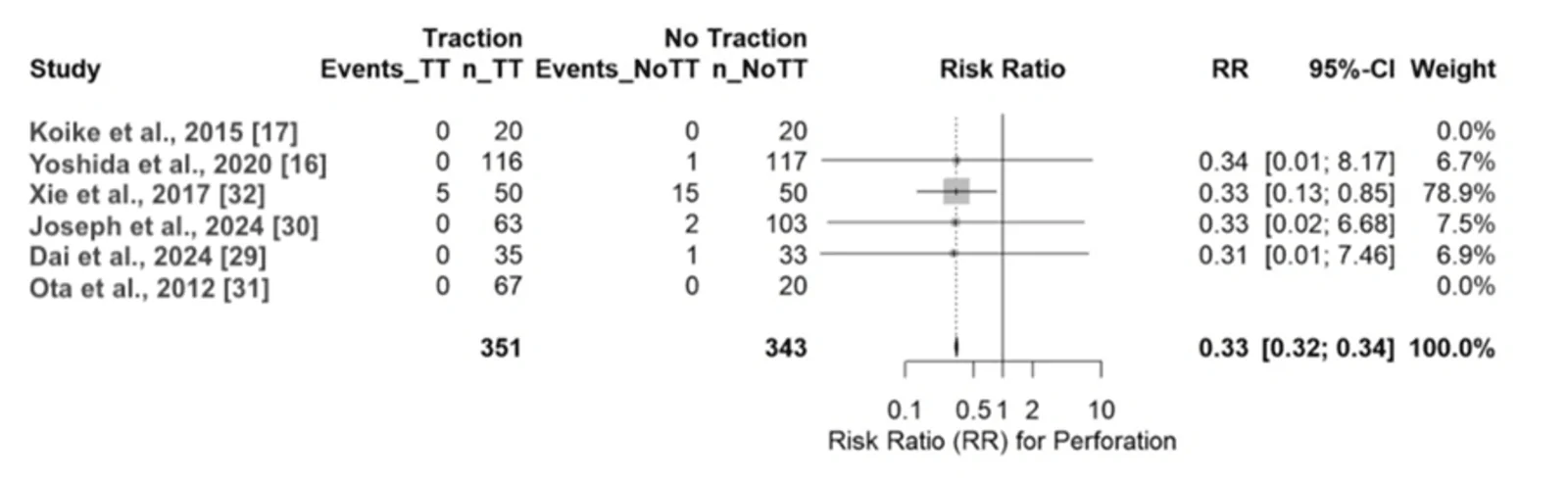
Forest plot, RR of perforation rates of TA-ESD vs C-ESD in esophageal cancer TA-ESD: Traction-assisted endoscopic submucosal dissection; C-ESD: Conventional endoscopic submucosal dissection.

Perforation rates: The pooled perforation rates associated with TA-ESD and C-ESD were 2.0% (CI = 0.5-7.2; I^2^ = 51.9%) and 3.6% (CI = 0.7-17.2; I^2^ = 85%), respectively, with RR 0.55 (CI = 0.32-0.94; p = 0.03) across five studies. The corresponding forest plots for RR are shown in Figure 6.

Mediastinitis: Mediastinitis as a complication had a pooled rate of occurrence of 0.7% (CI = 0.1-3.3; I^2^ = 0%) among the TA-ESD group and 1.1% (CI = 0.3-4.4; I^2^ = 0%) among the C-ESD group, with RR 0.34 (0.014-8.17; p = 0.5).

Pneumonia: Two studies reported this outcome. There was no reported case of pneumonia in the TA-ESD and C-ESD groups.

Postoperative bleeding: The postoperative bleeding rates in the TA-ESD and C-ESD groups were 2.1% (CI = 0.9-5.0; I^2^ = 0%) and 2.2% (CI = 0.9-5.2; I^2^ = 0%), with RR 0.89 (0.18-4.48; p = 0.79).

**Figure 7:**
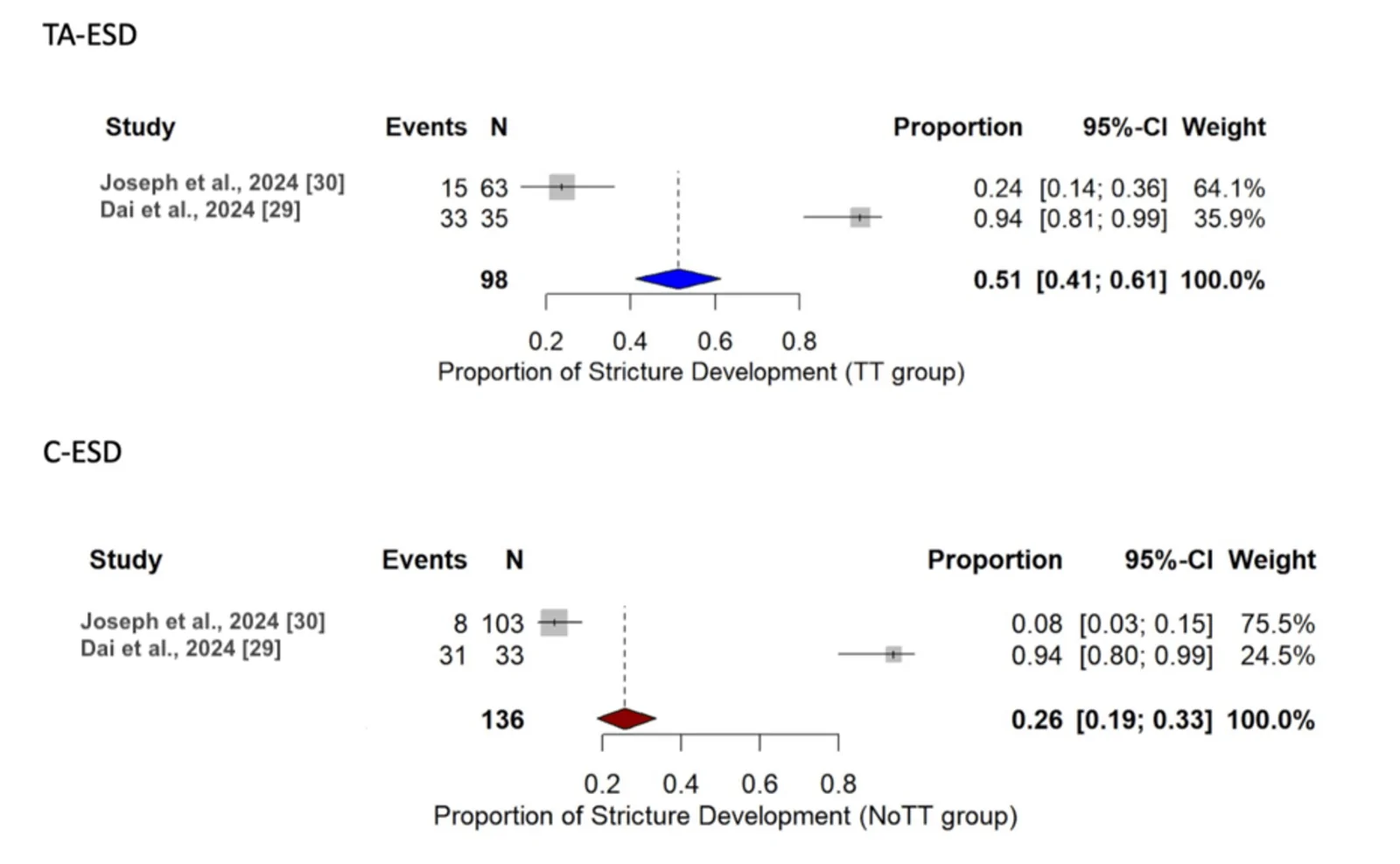
Forest plot, pooled rates of stricture formation rates of TA-ESD vs C-ESD in esophageal cancer TA-ESD: Traction-assisted endoscopic submucosal dissection; C-ESD: Conventional endoscopic submucosal dissection.

**Figure 8:**
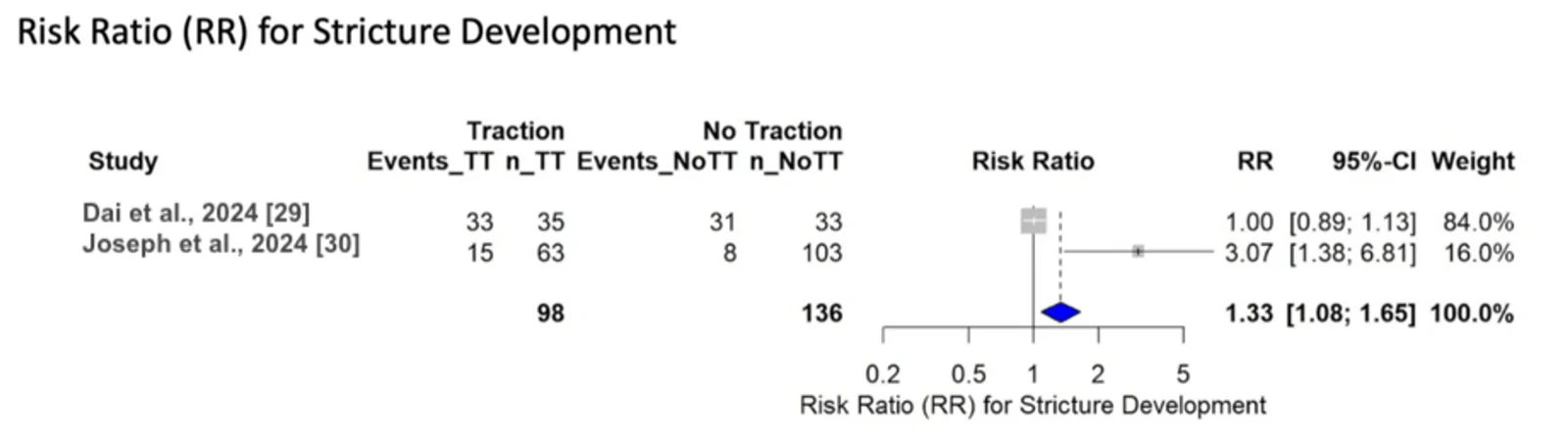
Forest plot, RR of stricture formation rates of TA-ESD vs C-ESD in esophageal cancer TA-ESD: Traction-assisted endoscopic submucosal dissection; C-ESD: Conventional endoscopic submucosal dissection.

Stricture formation: Two studies reported stricture formation. The pooled rates of stricture development with the intervention were statistically higher with TA-ESD, 35.4% (CI = 24.3-48.4; I^2^ = 96.1%), in comparison to C-ESD, 19.5% (CI = 11.3-31.6; I^2^ = 97.5%), with RR 1.33 (CI = 1.08-1.65; p = 0.0079). The corresponding forest plots for pooled rates and RR are shown in Figures 7, 8, respectively.

Recurrence rates: The recurrence rates associated with TA-ESD and C-ESD were 2.9% (CI = 1.0-17.7; I^2^ = 0%) and 6.1% (CI = 1.5-21.2; I^2^ = 0%), respectively, with RR 0.47 (0.04-4.95; p = 0.53), indicating that the result was statistically insignificant. The results are summarized in Table 3.

**Table 3:** Summary of pooled rates TA-ESD: Traction-assisted endoscopic submucosal dissection; C-ESD: Conventional endoscopic submucosal dissection.

Outcomes	Pooled proportions (95% confidence interval); I^2^%		Pooled RR, p-value
	TA-ESD	C-ESD	
En-bloc resection	96.9% (90.9-98.7); I² = 19.3%	96.8% (82.5-99.5); I² = 74.6%	1.01 (0.96-1.09); p = 0.68
R0 resection	86.6% (62.3-96.2); I² = 89.5%	77.6% (42.9-94.1); I² = 95%	1.11 (0.81-1.51); p = 0.28
Curative resection	52.5% (42.4-62.4); I² = 97.8%	36.6% (25.4-49.5); I² = 98.2%	1.46 (1.15-1.84); p = 0.0018
Total adverse events	0.9% (0.2-3.6); I² = 0%	4.1% (2.0-8.2); I² = 0%	0.08 (0.004-1.36); p = 0.08
Perforation	2.0% (0.5-7.2); I² = 51.9%	3.6% (0.7-17.2); I² = 85%	0.55 (0.32-0.94); p = 0.03
Postoperative bleeding	2.1% (0.9-5.0); I² = 0%	2.2% (0.9-5.2); I² = 0%	0.89 (0.18-4.48); p = 0.79
Mediastinitis	0.7% (0.1-3.3); I² = 0%	1.1% (0.3-4.4); I² = 0%	0.34 (0.014-8.17); p = 0.5
Stricture development	35.4% (24.3-48.4); I² = 96.1%	19.5% (11.3-31.6); I² = 97.5%	1.33 (1.08-1.65); p = 0.0079
Recurrence	2.9% (1.0-17.7); I² = 0%	6.1% (1.5-21.2); I² = 0%	0.47 (0.04-4.95); p = 0.53
Mean age (years)	67.97 ± 4.04	68.32 ± 3.14	
Mean Tumor diameter (mm)	32.32 (24.0-42.8)	33.1 (26.4-44.2)	
Mean Dissection time (mins)	71.48 ± 49.63	72.02 ± 31.52	
Mean resection area (cm^2^)	32.49 (21.98-43.0)	25.05 (24.0-26.1)	

Validation of meta-analysis results

Sensitivity Analysis

To evaluate whether any single study disproportionately influenced the meta-analysis, we conducted sensitivity analyses by sequentially omitting each study and reassessing the overall summary estimate. This approach revealed that no individual study had a significant impact on the primary outcomes or the observed heterogeneity. We also performed subgroup analyses stratified by study design, such as retrospective studies.

Heterogeneity

Heterogeneity varied substantially by outcome type. Safety outcomes generally showed low heterogeneity (I^2^ = 0% for most), whereas several efficacy outcomes, such as R0 resection, curative resection, and stricture development, showed substantial to extreme heterogeneity (I^2^ > 75% and >96% for curative resection and stricture development specifically). This variability likely reflects differences in patient case mix (depth of invasion and lesion size), technique, and operator experience rather than a single dominant source and is discussed further in the following section. Subgroup analysis was limited by the small number of studies.

Publication Bias

Publication bias was not formally assessed given fewer than 10 studies contributed to any single outcome.

Discussion 

ESD has been considered the first-line treatment for superficial esophageal cancers. Studies have shown that TA-ESD is a more efficient procedure, resulting in shorter durations and fewer adverse effects. Our meta-analysis evaluated the clinical outcomes comparing TA-ESD and C-ESD in 694 subjects from six studies. We report that TA-ESD demonstrated very similar dissection times to C-ESD. The resection area was slightly greater with TA-ESD compared to C-ESD (32.49 vs 25.05 cm^2^). Moreover, TA-ESD was associated with significantly better curative resection rates with a lower incidence of perforation. However, pooled rates of stricture development were higher with TA-ESD compared to C-ESD. These findings highlight the fact that neither approach is universally optimal, and both approaches have downsides.

In our meta-analysis, TA-ESD and C-ESD had almost the same dissection time. Nunes et al. [10] and Su et al. [15] in their meta-analysis showed that TA-ESD was associated with shorter dissection time (Su et al.: mean difference = 16.02; CI = - 22.71 to - 9.33 and Nunes et al.: mean difference = 10.75; CI = -18.76 to -2.74; p = 0.009).

Our study showed that the mean resected area was slightly greater with TA-ESD compared to C-ESD (32.49 vs 25.05 cm^2^). However, only two studies reported this outcome. No prior meta-analysis has compared the resection area as an outcome. The increased resection area associated with TA-ESD is likely due to enhanced tissue exposure and mobilization, better dissection angles, triangulation of tissue helping in improved dissection precision, and minimized tissue compression [33,34]. Our analysis reports no statistically significant difference in TA-ESD and C-ESD en-bloc and R0 resection rates, 96.9% vs 96.8 (p = 0.68) and 86.6% vs 77.6% (p = 0.28), respectively. Nunes et al., in their meta-regression, also reported no significant difference in en-bloc resection rates (relative difference: <0.00; CI = -0.01 to 0.01; p = 0.55) [10].

Our study showed that TA-ESD was associated with significantly higher curative resection rates compared to C-ESD, 52.5% vs 36.6% (p = 0.0018). No prior meta-analysis has compared this outcome. The higher curative resection rates suggest that TA-ESD (which has shorter operative time) would be a more cost-effective treatment intervention, especially for superficial esophageal cancers. However, this estimate must be interpreted with considerable caution: it derives from only two contributing studies with markedly different patient populations. Joseph et al. enrolled predominantly T1b/deeper-invasion lesions, with baseline curative resection rates of 9%-25%, while Dai et al. enrolled circumferential superficial (largely mucosal) lesions, with baseline curative resection rates of 85%-94% [29,30]. As outlined in the “Introduction” section, curability by endoscopic resection tracks closely with depth of invasion; the extreme heterogeneity we observed (I^2^ > 97% in both arms) is therefore very plausibly a case-mix artifact rather than evidence that TA-ESD itself confers a curative-resection advantage. We present this finding as hypothesis-generating rather than definitive and recommend that future studies stratify curative resection by depth of invasion before pooling across TA-ESD and C-ESD arms.

Furthermore, although clinically insignificant, the amount of local injection used (in mL) was less with TA-ESD compared to C-ESD, 20.30 ± 25.05 vs 27.31 ± 28.02 mL, as reported by Yoshida et al. and Koike et al. [16,17]. Yoshida et al. and Koike et al. reported that TA-ESD provided an improved field of vision and dissection process requiring fewer local injections [16,17]. Moreover, in C-ESD, the weight of the lesion caused the submucosal layer to collapse, and the injected fluid drained out, making it harder to keep the area distended and the view clear [16].

With regard to safety, no significant differences in total adverse events, postoperative bleeding, or pneumonia/mediastinitis were observed. However, the result for adverse events was approaching statistical significance, with TA-ESD associated with lower adverse events compared to C-ESD (0.9% vs 4.1%, p = 0.08). Nunes et al. also showed similar results in the esophageal cancer subgroup [10]. Su et al. demonstrated significantly lower adverse event rates and fewer perforations with TA-ESD for esophageal cancer, but their esophageal subgroup sample size was small. The most common adverse effect after ESD is stricture development [35,36]. Perforation rates were lower with TA-ESD compared to C-ESD (2% vs 3.6%, p = 0.03). This may be attributed to improved visualization of the submucosal layer. By maintaining tension on the lesion, TA-ESD minimizes tissue collapse and clarifies dissection planes, allowing precise identification of the submucosal fibers and avoidance of deeper cuts into the muscularis propria [16,33]. Moreover, shorter procedural times (though not statistically significant in our analysis) may contribute. Prior studies suggest that prolonged dissection increases edema and tissue friability, elevating perforation risk [11,15]. Another contributing factor may be reduced reliance on electrosurgical knife pressure. In C-ESD, endoscopists often apply downward force to stabilize the lesion, increasing the risk of transmural thermal injury or mechanical perforation. In contrast, traction methods (e.g., clip-and-thread or internal traction devices) provide external stabilization, permitting lighter instrument contact and safer dissection [17,34]. However, it is important to note that moderate to substantial heterogeneity in perforation rates in our study points toward variability in techniques across the studies, warranting standardization to optimize outcomes.

In our study, stricture development was more frequently seen with TA-ESD (35.4% vs 19.5%, p = 0.0079). However, only two studies reported this outcome, and hence the findings require careful contextual interpretation. Stricture formation after esophageal ESD is primarily driven by circumferential extension of mucosal resection and lesion length, and not necessarily the technique used for dissection [35-37]. In our study, TA-ESD was associated with a large resection area; the higher stricture rates likely reflect that traction-assisted methods were applied preferentially to larger, more circumferential lesions rather than representing a direct adverse effect of traction itself. Joseph et al. linked increased strictures in the TA-ESD group to a wider resected area, suggesting TA-ESD for larger lesions [30]. Dai et al. also found more strictures with TA-ESD but noted good response to on-demand endoscopic dilation and long-term favorable outcomes [29]. Further studies should stratify stricture rates by circumferential extent to distinguish whether stricture formation is associated with lesion characteristics or technique effects.

Strengths and Limitations

This review has several strengths: it is, to our knowledge, the first meta-analysis focused specifically on TA-ESD versus C-ESD for esophageal cancer, uses the most up-to-date literature as per our knowledge, and applies sensitivity and subgroup analyses despite a limited evidence base.

Our study has limitations. Including observational studies, such as retrospective analyses, with randomized controlled trials may have led to selection bias and confounding factors. Moreover, some studies did not report all relevant outcomes. Some results were reported by as few as two studies, and hence the pooled estimates for those reported outcomes are highly susceptible to being driven by a single study. Moreover, ESD is a practice-driven and endoscopist experience-dependent technique. Not all the studies reported endoscopist experience, and hence our analysis was unable to identify a clear threshold for how the experience of the endoscopist influences the outcomes. This likely introduces performance bias and impacts the results. Different traction methods were used in the studies analyzed, but due to small sample size, subgroup analysis was deferred. Moreover, significant heterogeneity in the results warrants caution in interpretation of results. This can be due to fewer studies with limited patient populations. The small number of included studies precluded formal subgroup analysis or meta-regression to explore sources of heterogeneity such as lesion size, circumferential extent, tumor location, invasion depth, fibrosis, and traction technique. Another limitation was the lack of uniformly reported data on important tumor-related variables that could influence the ESD outcomes, such as lesion size, tumor location, circumferential extent, invasion depth, and prior treatment. As a result, future prospective studies with larger sample sizes are needed. This review was not prospectively registered, and the English-language restriction may have excluded relevant non-English studies.

Although there are limitations, the updated literature review and analysis of the latest available data on esophageal cancer treatment highlight this article's strengths. To our knowledge, this is the most current meta-analysis reporting outcomes of TA-ESD versus C-ESD in the treatment of esophageal cancer. Moreover, some studies have compared TA-ESD and C-ESD for all gastrointestinal malignancies together, but their subgroup analysis for esophageal cancers is limited by their small sample size and limited number of outcomes compared.

## Conclusions

Our meta-analysis indicates that TA-ESD offers clear procedural and safety advantages over C-ESD for esophageal cancer treatment: comparable en-bloc and R0 resection rates, a larger resection area (32.49 vs 25.05 cm², p < 0.05), and a lower perforation rate (2.0% vs 3.6%, p = 0.03) - findings supported by lower heterogeneity and therefore the most robust in this analysis. TA-ESD was also associated with a higher pooled curative resection rate (52.5% vs 36.6%, p = 0.0018) and a higher rate of stricture formation (35.4% vs 19.5%, p = 0.0079); however, both of these estimates derive from only two studies with extreme heterogeneity driven largely by differences in lesion depth and size across the contributing cohorts, and should be regarded as hypothesis-generating rather than conclusive. TA-ESD's improved visualization and tissue stabilization may make it particularly valuable for larger or more complex lesions, while C-ESD remains a viable option for standard cases. Future large-scale prospective studies stratifying by depth of invasion and lesion size, comparing different traction techniques, and incorporating cost-effectiveness analysis are needed before firm treatment recommendations can be made. Until then, the choice between TA-ESD and C-ESD should be individualized, weighing TA-ESD's procedural and safety advantages against its associated stricture risk and the still-preliminary evidence on curative resection.

## References

[1] Draganov PV, Wang AY, Othman MO, Fukami N (2019). AGA institute clinical practice update: endoscopic submucosal dissection in the United States. Clin Gastroenterol Hepatol.

[2] Saito Y, Uraoka T, Yamaguchi Y (2010). A prospective, multicenter study of 1111 colorectal endoscopic submucosal dissections (with video). Gastrointest Endosc.

[3] Fujiyoshi Y, Khalaf K, He T (2024). Comparison of EMR versus endoscopic submucosal dissection for Barrett's neoplasia and esophageal adenocarcinoma: a systematic review and meta-analysis. Gastrointest Endosc.

[4] Ono S, Fujishiro M, Niimi K, Goto O, Kodashima S, Yamamichi N, Omata M (2009). Long-term outcomes of endoscopic submucosal dissection for superficial esophageal squamous cell neoplasms. Gastrointest Endosc.

[5] Onozato Y, Ishihara H, Iizuka H, Sohara N, Kakizaki S, Okamura S, Mori M (2006). Endoscopic submucosal dissection for early gastric cancers and large flat adenomas. Endoscopy.

[6] Oda I, Gotoda T, Hamanaka H (2005). Endoscopic submucosal dissection for early gastric cancer: technical feasibility, operation time and complications from a large consecutive series. Dig Endosc.

[7] Oyama T, Tomori A, Hotta K (2005). Endoscopic submucosal dissection of early esophageal cancer. Clin Gastroenterol Hepatol.

[8] Hazama H, Tanaka M, Kakushima N (2019). Predictors of technical difficulty during endoscopic submucosal dissection of superficial esophageal cancer. Surg Endosc.

[9] Okubo Y, Ishihara R (2023). Endoscopic submucosal dissection for esophageal cancer: current and future. Life (Basel).

[10] Nunes FG, Gomes IL, De Moura DT (2024). Conventional versus traction-assisted endoscopic submucosal dissection for esophageal, gastric, and colorectal neoplasms: a systematic review and meta-analysis of randomized controlled trials. Cureus.

[11] Bahdi F, Mercado MM, Huang X (2023). Prospective evaluation of a standardized approach to improve procedure speed in esophageal endoscopic submucosal dissection. Tech Innov Gastrointest Endosc.

[12] Odagiri H, Yasunaga H (2017). Complications following endoscopic submucosal dissection for gastric, esophageal, and colorectal cancer: a review of studies based on nationwide large-scale databases. Ann Transl Med.

[13] Maeda Y, Hirasawa D, Fujita N (2011). Mediastinal emphysema after esophageal endoscopic submucosal dissection: its prevalence and clinical significance. Dig Endosc.

[14] Bhatt A, Abe S, Kumaravel A, Vargo J, Saito Y (2015). Indications and techniques for endoscopic submucosal dissection. Am J Gastroenterol.

[15] Su YF, Cheng SW, Chang CC, Kang YN (2020). Efficacy and safety of traction-assisted endoscopic submucosal dissection: a meta-regression of randomized clinical trials. Endoscopy.

[16] Yoshida M, Takizawa K, Nonaka S (2020). Conventional versus traction-assisted endoscopic submucosal dissection for large esophageal cancers: a multicenter, randomized controlled trial (with video). Gastrointest Endosc.

[17] Koike Y, Hirasawa D, Fujita N (2015). Usefulness of the thread-traction method in esophageal endoscopic submucosal dissection: randomized controlled trial. Dig Endosc.

[18] Garg A, Moond V, Bassi M (2025). Conventional versus traction-assisted endoscopic submucosal dissection for esophageal cancers: a systematic review and meta-analysis. Gastroenterology.

[19] Stroup DF, Berlin JA, Morton SC (2000). Meta-analysis of observational studies in epidemiology: a proposal for reporting. Meta-analysis of observational studies in epidemiology (MOOSE) group. JAMA.

[20] Stang A (2010). Critical evaluation of the Newcastle-Ottawa scale for the assessment of the quality of nonrandomized studies in meta-analyses. Eur J Epidemiol.

[21] Cotton PB, Eisen GM, Aabakken L (2010). A lexicon for endoscopic adverse events: report of an ASGE workshop. Gastrointest Endosc.

[22] DerSimonian R, Laird N (1986). Meta-analysis in clinical trials. Controlled Clinical Trials.

[23] Higgins JP, Thompson SG, Spiegelhalter DJ (2009). A re-evaluation of random-effects meta-analysis. J R Stat Soc Ser A Stat Soc.

[24] Higgins JP, Thompson SG, Deeks JJ, Altman DG (2003). Measuring inconsistency in meta-analyses. BMJ.

[25] Riley RD, Higgins JP, Deeks JJ (2011). Interpretation of random effects meta-analyses. BMJ.

[26] Easterbrook PJ, Gopalan R, Berlin JA (1991). Publication bias in clinical research. Lancet.

[27] Luo D, Wan X, Liu J, Tong T (2018). Optimally estimating the sample mean from the sample size, median, mid-range, and/or mid-quartile range. Stat Methods Med Res.

[28] Wan X, Wang W, Liu J, Tong T (2014). Estimating the sample mean and standard deviation from the sample size, median, range and/or interquartile range. BMC Med Res Methodol.

[29] Dai N, Ullah S, Zhang J (2024). The efficacy and safety of snare traction-assisted endoscopic submucosal dissection for circumferential superficial esophageal cancer. Surg Endosc.

[30] Joseph A, Vantanasiri K, Draganov PV (2024). Endoscopic submucosal dissection with versus without traction for pathologically staged T1B esophageal cancer: a multicenter retrospective study. Gastrointest Endosc.

[31] Ota M, Nakamura T, Hayashi K (2012). Usefulness of clip traction in the early phase of esophageal endoscopic submucosal dissection. Dig Endosc.

[32] Xie X, Bai JY, Fan CQ (2017). Application of clip traction in endoscopic submucosal dissection to the treatment of early esophageal carcinoma and precancerous lesions. Surg Endosc.

[33] Lopimpisuth C, Simons M, Akshintala VS, Prasongdee K, Nanavati J, Ngamruengphong S (2022). Traction-assisted endoscopic submucosal dissection reduces procedure time and risk of serious adverse events: a systematic review and meta-analysis. Surg Endosc.

[34] Mitsuyoshi Y, Ide D, Ohya TR (2022). Training program using a traction device improves trainees' learning curve of colorectal endoscopic submucosal dissection. Surg Endosc.

[35] Martínek J, Juhas S, Dolezel R (2018). Prevention of esophageal strictures after circumferential endoscopic submucosal dissection. Minerva Chir.

[36] Kim GH, Jee SR, Jang JY (2014). Stricture occurring after endoscopic submucosal dissection for esophageal and gastric tumors. Clin Endosc.

[37] Bhatt A, Mehta NA (2020). Stricture prevention after esophageal endoscopic submucosal dissection. Gastrointest Endosc.

